# Direct percutaneous puncture of occluded venous stents as an adjunctive technique to restore patency

**DOI:** 10.1186/s42155-024-00514-x

**Published:** 2025-01-03

**Authors:** Gabriel E. Li, David S. Shin, Stephanie Sobrepera, Matthew Abad-Santos, Eric J. Monroe, Jeffrey Forris Beecham Chick

**Affiliations:** 1 Department of Radiology, Section of Vascular and Interventional Radiology, University of Washington, 1959 Northeast Pacific Street, Seattle, WA 98195 USA; 2 Department of Radiology, Division of Vascular and Interventional Radiology, University of Southern California, 1500 San Pablo Street, Los Angeles, CA 90033 USA; 3 Department of Radiology, University of Wisconsin, 1675 Highland Ave, Madison, WI 53792 USA

## Short communication

Venous stent occlusions represent a clinical challenge. Primary patency rates for venous stents, particularly in chronic venous diseases, ranges from 57 to 79% at 72-months [1]. A variety of endovascular blunt and sharp recanalization techniques have been described, but may ultimately fail [2–4]. This report describes direct percutaneous puncture of occluded venous stents and stent-grafts as an adjunctive technique to gain access into the occluded system and restore patency. Technical success was defined as successful direct percutaneous puncture of occluded venous stents with subsequent recanalization. Clinical success was defined as improvement in presenting venous obstruction symptoms. *Institutional review board approval was obtained before preparation of this report.*

**Fig. 1:**
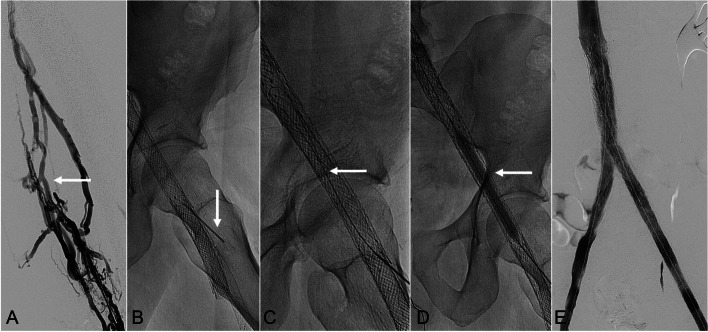
*41-year-old man with prior left iliocaval and iliofemoral stent reconstruction presenting with recurrent left lower extremity swelling in the setting of chronic stent thrombosis (>30-days)*. **A** Left lower extremity venography, from a left popliteal vein approach, showed chronic venous occlusive (post-thrombotic) changes throughout the left femoral veins (solid arrow). Endovascular recanalization of the occluded left iliofemoral stent construct was attempted, but was unsuccessful. **B** Under fluoroscopic-guidance, the occluded left iliofemoral Wallstent/Abre stent complex was punctured with an 18-gauge needle (solid arrow). **C** A Glidewire Hydrophilic Coated Guidewire (Terumno Medical Corpotation; Tokyo, Japan) (solid arrow) was advanced through the occluded left iliofemoral and iliocaval stent constructs. The wire was then snared via a right internal jugular vein access (not shown). **D** From the right internal jugular vein access, angioplasty of the occluded left iliofemoral stent construct was performed using an 8-mm balloon (solid arrow). The through-and-through wire access was removed, and a new wire access was established down the peripheral end of the stent construct and out to the inflow femoral vein. Prolonged angioplasty of the left femoral vein was performed (not shown). **E** Completion bilateral lower extremity venography demonstrated brisk in-line flow through both iliofemoral and iliocaval stent constructs as well as the inferior vena cava stent constructs

**Fig. 2:**
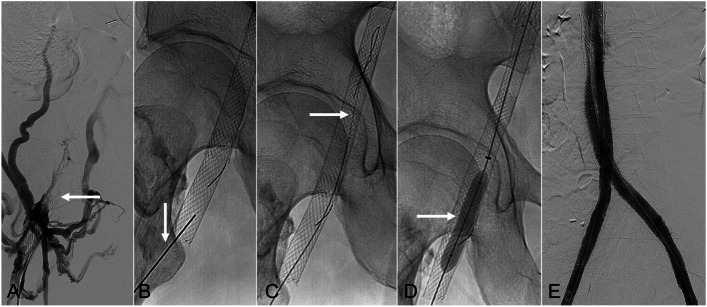
*62-year-old male with prior right iliocaval and iliofemoral stent reconstruction presenting with current right lower extremity swelling and pain in the setting of chronic stent thrombosis (>30-days).* **A** Right lower extremity venography, from a right femoral vein approach, showed the iliofemoral stent occlusion (solid arrow) with surrounding collateral vessels. Endovascular recanalization of the occluded right iliofemoral stent construct was attempted, but was unsuccessful. **B** Under fluoroscopic-guidance, the occluded right iliofemoral Wallstent was punctured with a 21-gauge needle (solid arrow). **C** A Nixtrex guidewire (Medtronic) (solid arrow) was advanced through the occluded right iliofemoral and iliocaval stent constructs. The wire was then snared via a right internal jugular vein access (not shown). **C** From the right internal jugular vein access, angioplasty of the occluded right iliofemoral stent construct was performed using an 8-mm balloon (solid arrow). Wire access was then established down to the inflow femoral vein, and prolonged venoplasty was performed (not shown). **E** Completion bilateral lower extremity venography demonstrated brisk in-line flow through both iliofemoral and iliocaval stent constructs as well as the inferior vena cava stent constructs

**Fig. 3:**
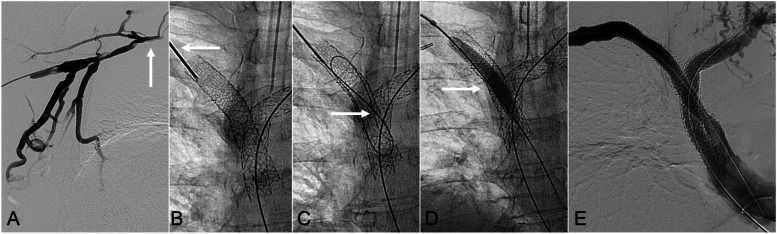
*61-year-old female with prior bilateral kissing brachiocephalocaval stent-graft reconstruction presenting with recurrent right upper extremity swelling and pain in the setting of chronic stent thrombosis (>30-days).* **A** Right upper extremity venography, from a right brachial vein approach, showed occlusion of the right subclavian vein (solid arrow) and brachiocephalocaval stent. Endovascular recanalization was attempted, but was unsuccessful. **B** Under ultrasound and fluoroscopic-guidance, the peripheral end of the occluded right brachiocephalocaval Viabahn stent-graft was punctured with an 18-gauge needle (solid arrow). **C** A Glidewire Hydrophilic Coated Guidewire (Terumno Medical Corpotation) (solid arrow) was advanced through the occluded brachiocephalocaval stent-graft. The wire was then snared via a right common femoral vein access (not shown). **D** From the right common femoral vein access, a wire was advanced through the thrombosed stent-graft then out to a right brachial vein Angioplasty with an 8-mm balloon (solid arrow) was performed to restore a patent channel. An additional Viabahn stent-graft was placed to extend the construct to the subclavian vein. **E** Completion bilateral upper extremity venography demonstrated brisk in-line flow through both brachiocephalocaval stent-graft constructs

Four patients, including two (50%) males and two (50%) females (mean age: 43.3 ± 19.5 years; range: 17–59 years), underwent percutaneous puncture of occluded venous stents as an escalation technique during the recanalization procedure. Mean time from initial stent placement to intervention was 344 ± 174 days (range: 229–602 days). Presenting symptoms included swelling in three (75%), pain in two (50%), erythema in one (25%), and exercise intolerance in one (25%) patient. Pre-procedure Doppler ultrasound imaging was obtained in all four (100%) patients, and one (25%) patient underwent additional computed tomographic venography. All (*n* = 4; 100%) patients with occluded venous stents had failed same session conventional endovascular blunt and sharp (back end of wire) recanalization techniques from both antegrade and retrograde approaches. Occluded stents were accessed using an 18-gauge needle in three (75%) patients and a 21-gauge needle in one (25%) patient. Wires were then advanced, snared from a distant access to obtain-through-and-through venous access, to facilitate angioplasty and stent reconstructions. *Direct percutaneous puncture technique is shown in* Figs. 1, 2 and 3. Occluded stents were accessed at the subclavian (*n* = 1; 25%), external iliac (*n* = 1; 25%), common femoral (*n* = 1; 25%), and femoral (*n* = 1; 25%) veins. The accessed stents included Wallstent Endoprostheses (Boston Scientific, Marlborough, MA, USA) in two (50%) patients, a Wallstent Endoprosthesis (Boston Scientific) relined with Abre Venous Stent (Medtronic; Dublin, Ireland) in one (25%), and Viabahn Endoprosthesis (Gore Medical, Newark, DE, USA) in one (25%) patient. Mean diameter of the accessed stents was 12.0 ± 1.6-mm (range: 10–14-mm). Technical and clinical successes were achieved in all (*n* = 4; 100%) patients. There were no adverse events related to the direct puncture of the stents. Additional stent placement was performed in two (50%) patients. Mean fluoroscopy time for the recanalization procedures was 75.7 ± 46.9 min (range: 21.6–105.0 min). Mean radiation dose was 1836.3 ± 1171.4 mGy (range: 597–3334 mGy). Mean total follow-up time from procedure was 1078 ± 573 days (range: 464–1687 days).

Although many chronic venous stent occlusions may be recanalized with conventional endovascular techniques, there are descriptions of failures requiring escalation techniques [2–4]. Sharp recanalization techniques, using a variety of devices, including the back ends of wires, radiofrequency devices, transseptal needles, or transjugular intrahepatic portosystemic shunt access sets, may also fail [2–4]. In the largest sharp recanalization series of 123 patients, 20 patients had chronic venous stent occlusions, three of which could not be recanalized [2]. In a series of radiofrequency wire recanalization of chronic venous stent occlusions, there were two technical failures [4]. The current series demonstrates an alternative escalation technique of direct percutaneous puncture of occluded venous stents to facilitate rapid intraluminal access when conventional endovascular techniques were unsuccessful. This technique, which utilizes ultrasound or fluoroscopy to guide direct puncture, bypasses chronic fibrous occlusions (“caps”) which are often found at the ends of the chronically occluded venous stents. Similar techniques have been used for recanalization of occluded femoropopliteal artery stents [5]. Such access may facilitate subsequent wire advancement through the stent interstices, into the thrombosed lumen, then out the inflow or outflow veins, which may be used to initiate subsequent recanalization techniques. While additional reports are warranted to establish safety, the direct puncture technique is feasible and useful as an escalation technique to facilitate recanalization of chronically occluded venous stents.

## Data Availability

Data for this study available upon reasonable request.
